# Supplementary material for: Shedding of N-acetylglucosaminyltransferase-V is regulated by maturity of cellular N-glycan
Source: Commun Biol. 2022 Aug 1;5:743. doi: 10.1038/s42003-022-03697-y (PMC9343384; doi:10.1038/s42003-022-03697-y)
Supplement: Supplementary file 2 — Supplementary Information [file 42003_2022_3697_MOESM2_ESM.pdf]

Supplementary Figures

Shedding of *N*-acetylglucosaminyltransferase-V is regulated by maturity of cellular *N*-glycan

Tetsuya Hirata[1], Misaki Takata[2], Yuko Tokoro[1], Miyako Nakano[2] and Yasuhiko Kizuka[1*]

[1]Institute for Glyco-core Research (iGCORE), Gifu University, Gifu 501-1193, Japan
[2]Graduate School of Integrated Sciences for Life, Hiroshima University, Higashihiroshima 739-8530, Japan

Supplementary Figure 1-11

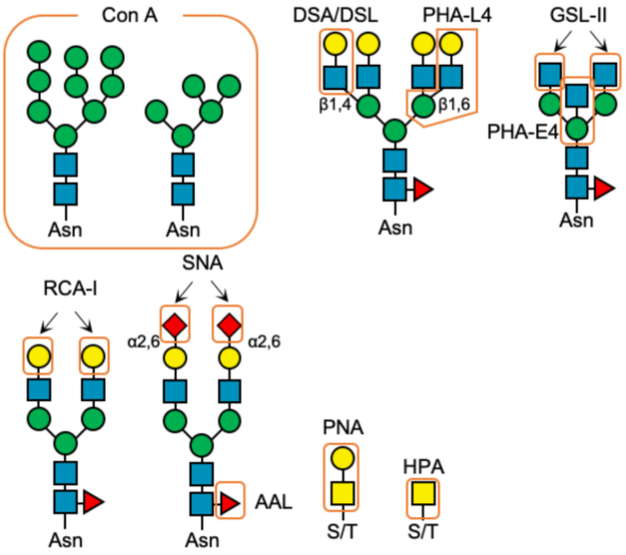

Supplementary Figure 1. Specificity of the lectins used in this study
Schematics indicate the glycan structures preferably bound by lectins used in this study.

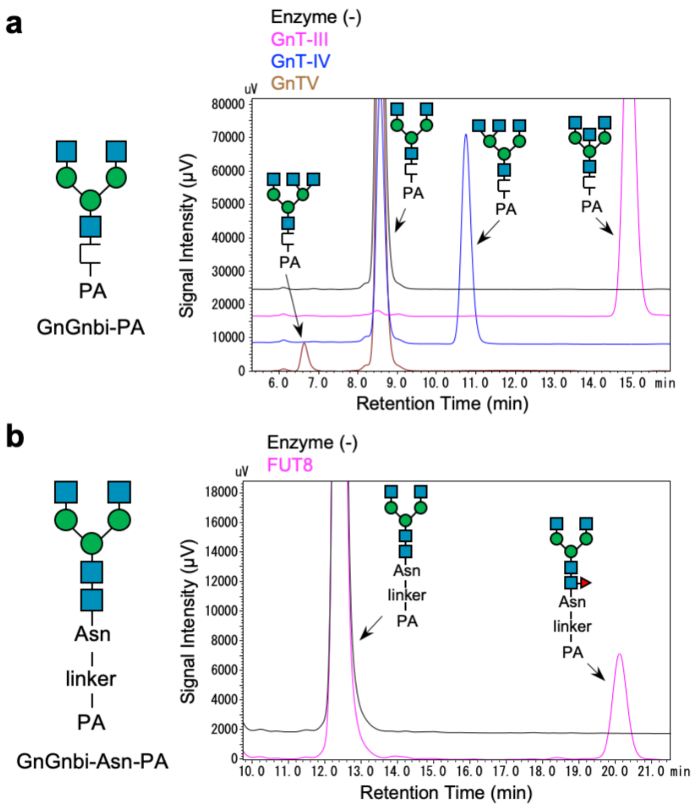

Supplementary Figure 2. ***In vitro*** **enzyme reactions using purified recombinant enzymes.**
a. Purified His-tagged GnT-III, -IVa, and -V were incubated with acceptor substrate GnGnbi-PA. The reaction mixtures were analyzed by reverse-phase HPLC. **b**. Purified His-tagged FUT8 was incubated with acceptor GnGnbi-Asn-PNS. The reaction mixture was analyzed by reverse-phase HPLC.

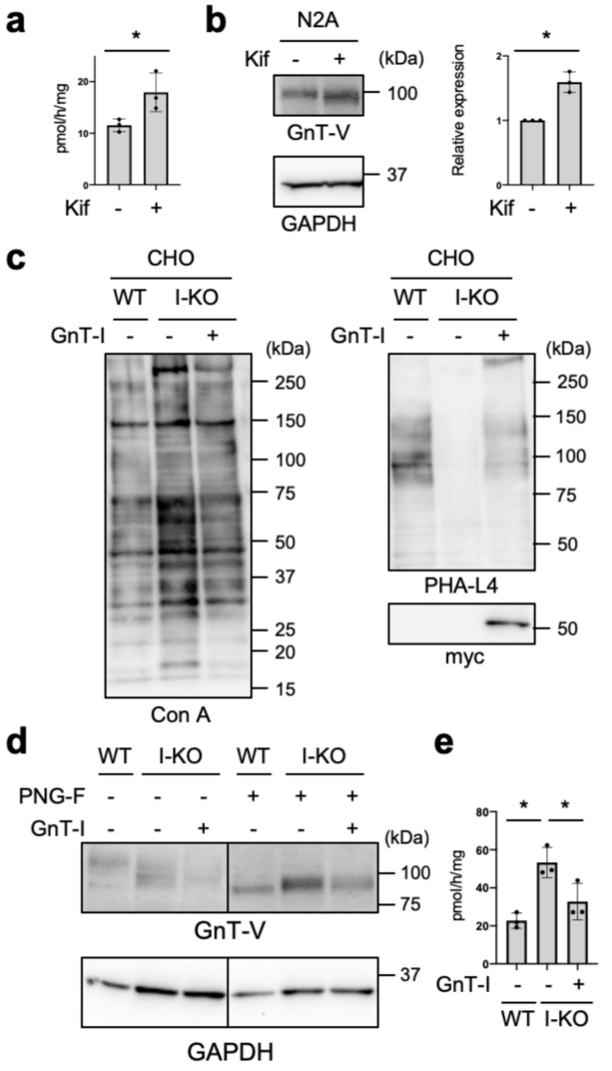

Supplementary Figure 3. Protein level and activity of GnT-V in Neuro-2A and CHO cells with altered cellular *N*-glycan structures.

a. The cellular activity of GnT-V in Neuro-2A cells treated with or without kifunensine (kif). Error bars represent SD ($n = 3$). Statistical analysis was by Welch's *t*-test. **b**. Proteins from Neuro-2A cells treated with or without kifunensine were blotted for GnT-V or GAPDH. The graph shows quantification of the GnT-V signals in western blots. Error bars represent SD ($n = 3$). Statistical analysis was by Welch's *t*-test. **c**. Proteins from WT, GnT-I-deficient (I-KO), and GnT-I (myc-tagged)-rescued CHO cells were blotted with Con A, PHA-L4, or anti-myc antibody. **d**. Proteins from WT, I-KO, and GnT-I-rescued CHO cells treated with or without PNGase F (PNG-F) were blotted with anti-GnT-V or anti-GAPDH antibodies. **e**. The cellular activity of GnT-V. Error bars represent SD ($n = 3$). Statistical analysis was by one-way ANOVA with *post-hoc* Tukey test. *$p < 0.05$.

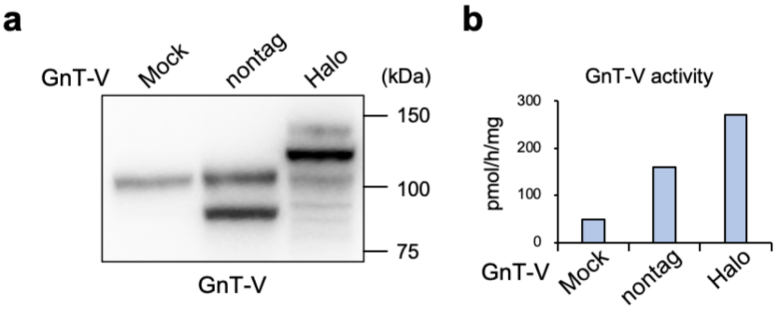

Supplementary Figure 4. Activity of Halo-tagged GnT-V.

a. hGnT-V WT and Halo-tagged hGnT-V were expressed in B16 cells and analyzed by western blotting with anti-GnT-V antibody. **b**. The GnT-V activity in B16 cells expressing hGnT-V WT and Halo-tagged hGnT-V was measured by *in vitro* enzyme assays.

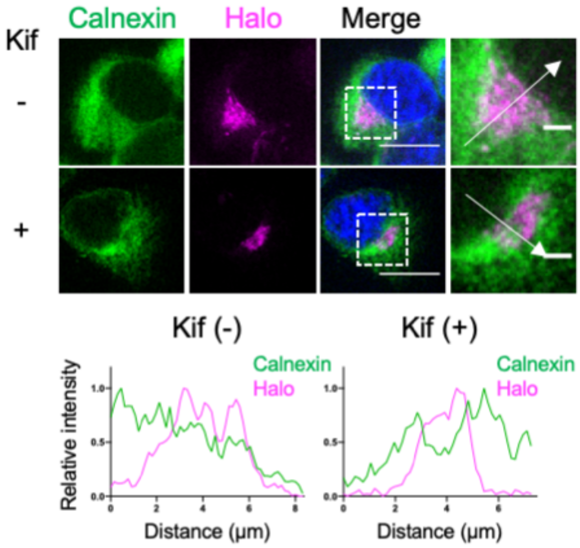

Supplementary Figure 5. Localization of GnT-V-Halo (Halo) in B16 cells treated with or without kifunensine.

(Upper) Calnexin was stained as an ER marker. (Lower) Line plots of relative fluorescence intensity are shown. Arrows indicate the plotted regions. Scale bars: 10 or 2 µm.

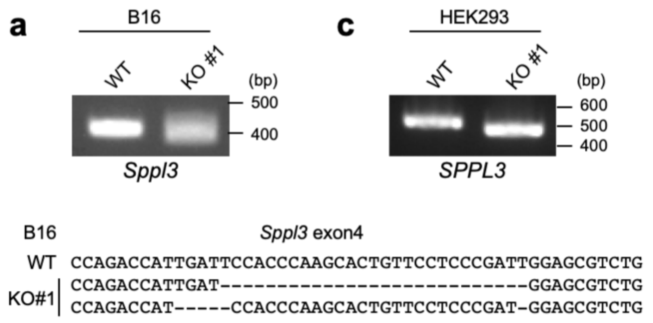

Supplementary Figure 6. KO validation of *SPPL3* genes in B16 cells and HEK293 cells.

a. Genomic DNA was extracted from WT or SPPL3-KO (clone #1) B16 cells, and DNA fragments including the target sequence of gRNAs were amplified by PCR. **b**. Sequence of genomic DNA around the target site of gRNAs from WT and the target allele of SPPL3-KO cells. **c.** Genomic DNA was extracted from WT or SPPL3-KO (clone #1) HEK293 cells, and DNA fragments including the target sequence of gRNAs were amplified by PCR.

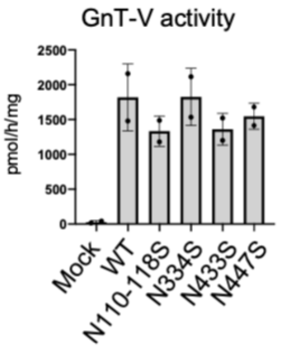

Supplementary Figure 7. Activities of GnT-V mutants lacking specific *N*-glycosylation sites. GnT-V WT or its mutants lacking specific *N*-glycosylation sites were expressed in HEK293 cells, and the GnT-V activities were measured by *in vitro* enzyme assay. N110-118S is a mutant in which residues N110, N115, and N118 are all mutated to serine.

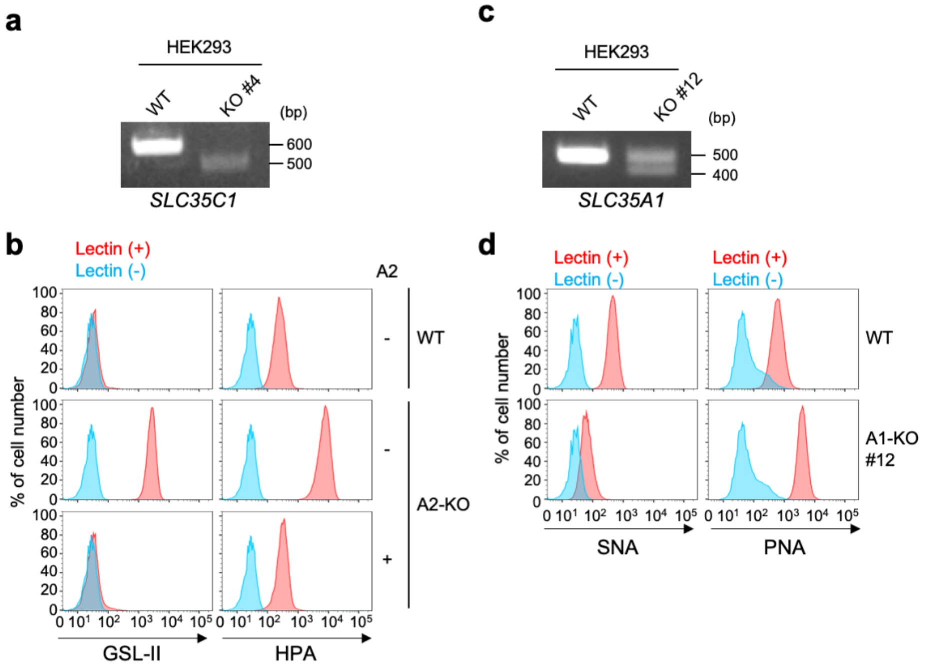

Supplementary Figure 8. KO validation of *SLC35C1*, *SLC35A2*, and *SLC35A1* in HEK293 cells. **a**. Genomic DNA was extracted from WT or SLC35C1-KO (clone #4) HEK293 cells, and DNA fragments including the target sequence of gRNAs were amplified by PCR. **b**. WT, SLC35A2-KO (A2-KO), or SLC35A2-rescued HEK293 cells were stained with GSL-II and HPA that bind to terminally exposed GlcNAc and GalNAc, respectively. **c**. Genomic DNA was extracted from WT or SLC35A1-KO (clone #12) HEK293 cells, and DNA fragments including the target sequence of gRNAs were amplified by PCR. **d**. WT, SLC35A1-KO (A1-KO), or SLC35A1-rescued cells were stained with SNA and PNA that essentially bind to α2,6-linked sialic acid and the Gal–GalNAc structure of *O*-glycans, respectively.

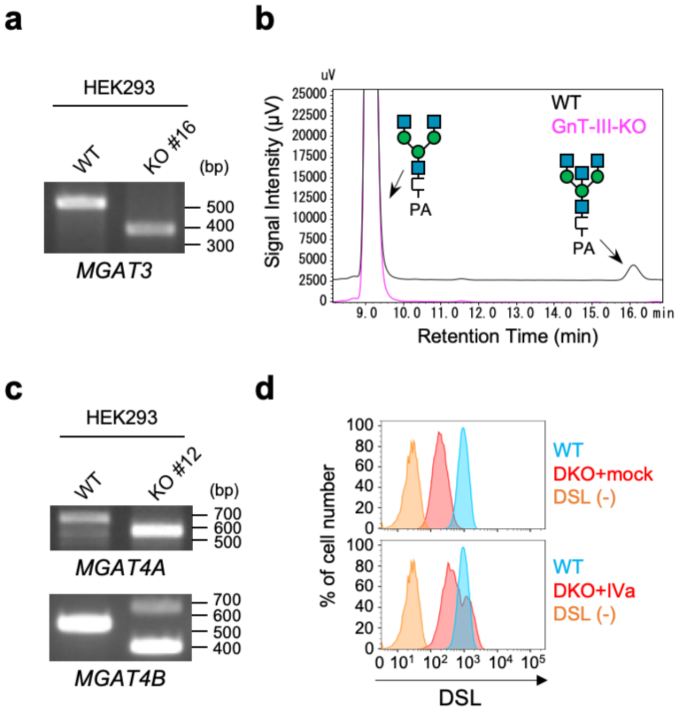

Supplementary Figure 9. KO validation of *MGAT3*, *MGAT4A*, and *MGAT4B* in HEK293 cells.
a. Genomic DNA was extracted from WT or GnT-III-KO (clone #16) HEK293 cells, and DNA fragments including the target sequence of gRNAs were amplified by PCR. *MGAT3*: the gene encoding GnT-III. **b**. GnT-III activity was measured in WT or GnT-III-KO cell lines. **c**. Genomic DNA was extracted from WT or GnT-IVa and IVb-DKO (clone #12) HEK293 cells, and DNA fragments including the target sequences of gRNAs were amplified by PCR. *MGAT4A*: the gene encoding GnT-IVa. *MGAT4B*: the gene encoding GnT-IVb. **d**. GnT-IVa and IVb-DKO and GnT-IVa-rescued HEK293 cells were stained with DSA, which binds to the β1,4-GlcNAc branch.

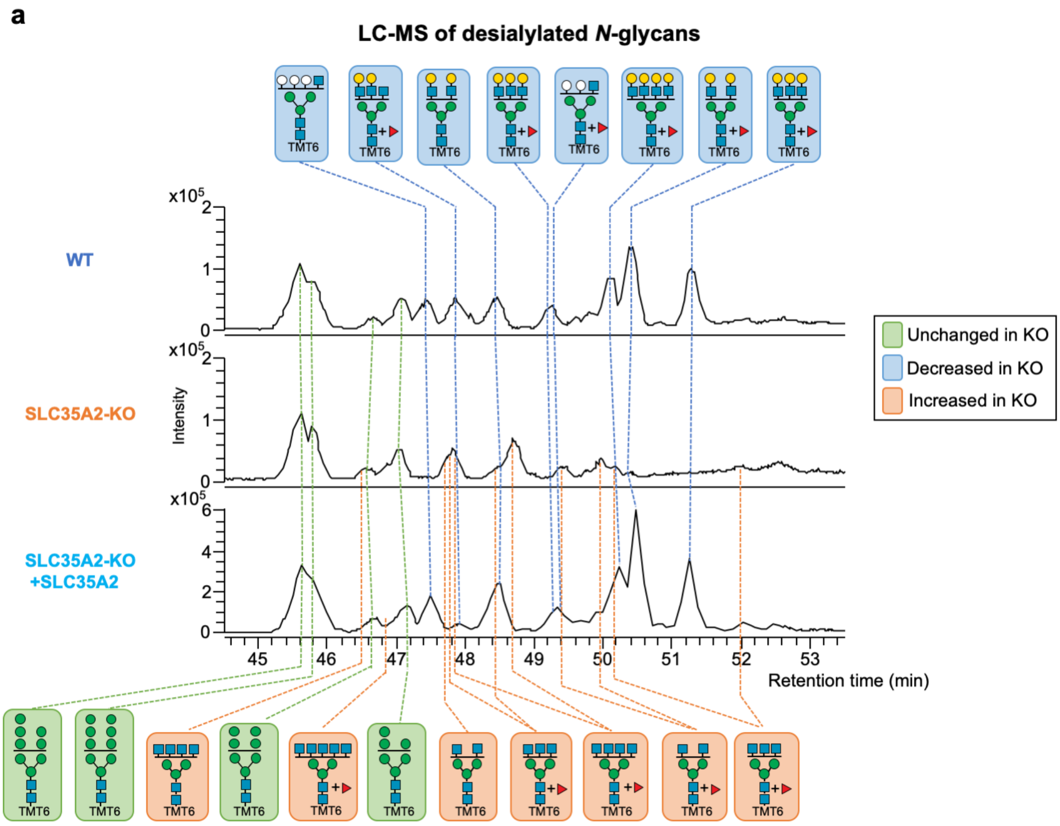
a
LC-MS of desialylated N-glycans
WT
SLC35A2-KO
SLC35A2-KO
+SLC35A2
Intensity
x10^5
Retention time (min)
Unchanged in KO
Decreased in KO
Increased in KO
TMT6

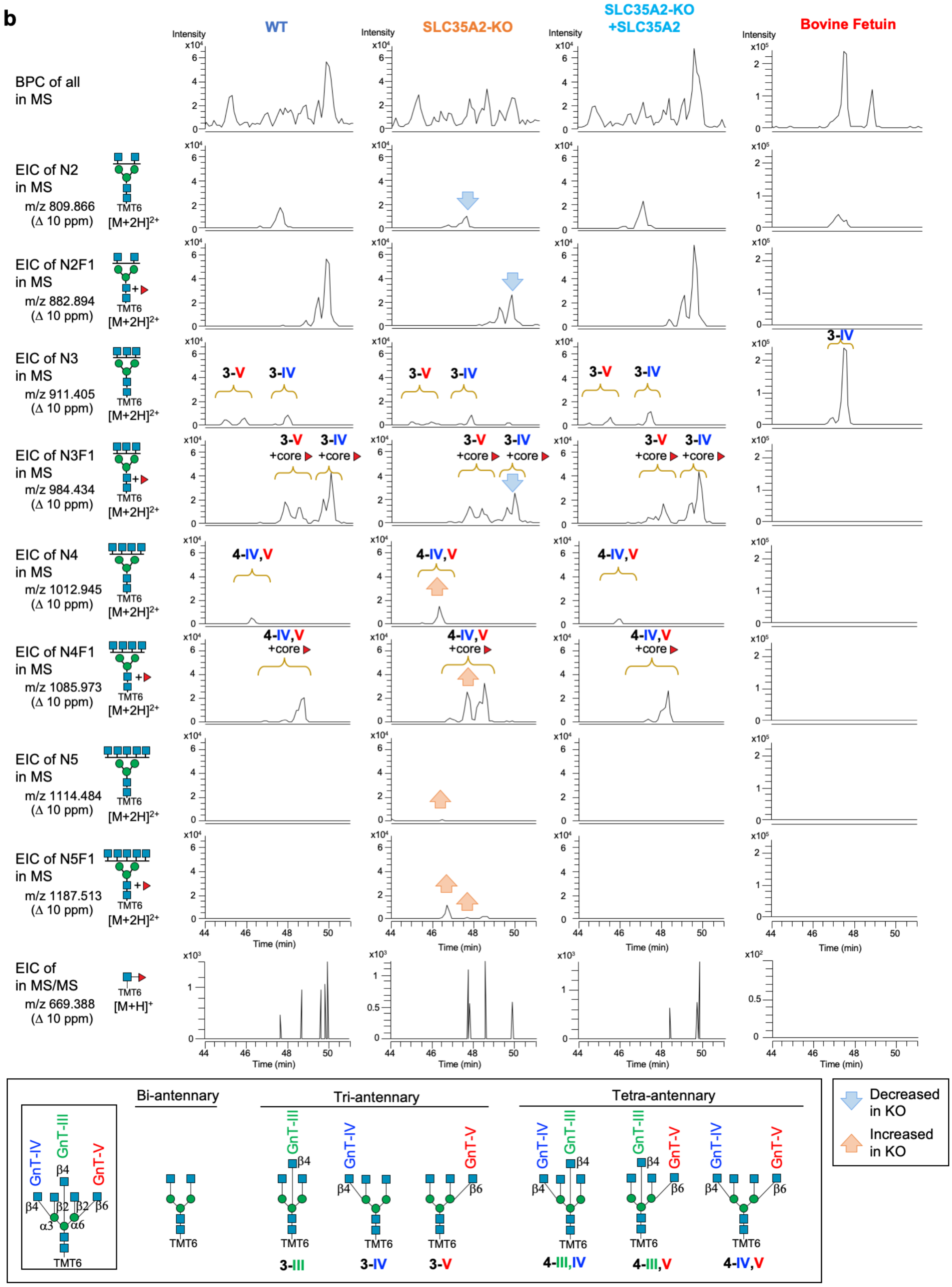
b
WT
SLC35A2-KO
SLC35A2-KO +SLC35A2
Bovine Fetuin
BPC of all in MS
EIC of N2 in MS m/z 809.866 (Δ 10 ppm) [M+2H]2+
EIC of N2F1 in MS m/z 882.894 (Δ 10 ppm) [M+2H]2+
EIC of N3 in MS m/z 911.405 (Δ 10 ppm) [M+2H]2+
EIC of N3F1 in MS m/z 984.434 (Δ 10 ppm) [M+2H]2+
EIC of N4 in MS m/z 1012.945 (Δ 10 ppm) [M+2H]2+
EIC of N4F1 in MS m/z 1085.973 (Δ 10 ppm) [M+2H]2+
EIC of N5 in MS m/z 1114.484 (Δ 10 ppm) [M+2H]2+
EIC of N5F1 in MS m/z 1187.513 (Δ 10 ppm) [M+2H]2+
EIC of in MS/MS m/z 669.388 (Δ 10 ppm) [M+H]+
Intensity
Time (min)
3-V
3-IV
+core
4-IV,V
TMT6
Bi-antennary
Tri-antennary
Tetra-antennary
GnT-IV
GnT-III
GnT-V
3-III
4-III,IV
4-III,V
Decreased in KO
Increased in KO

c

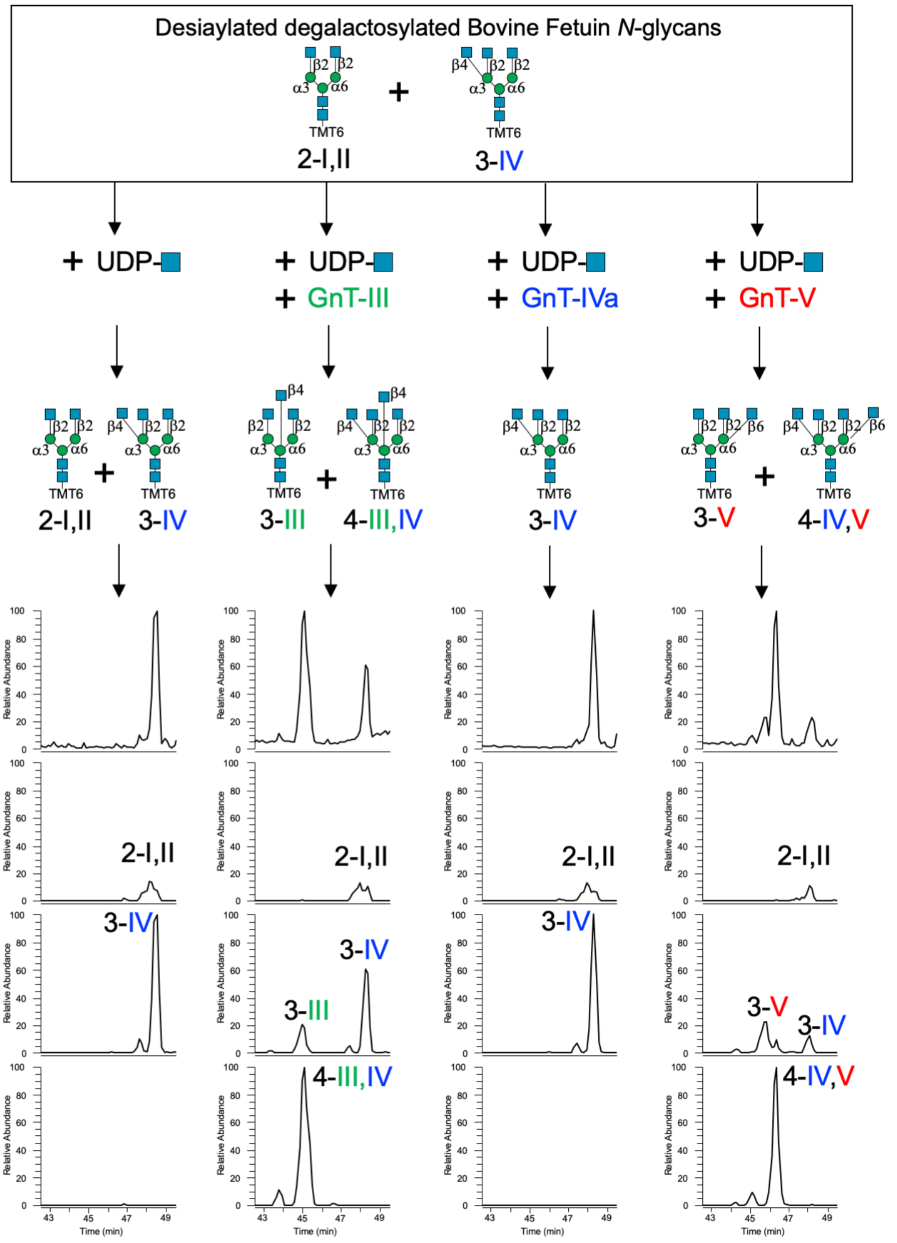

Supplementary Figure 10. *N*-Glycomics of WT, SLC35A2-KO, and SLC35A2-rescued HEK293 cells.

a. Base peak chromatogram (BPC) from LC-ESI MS analysis of TMT6-labeled desialylated *N*-glycans from WT, SLC35A2-KO (A2-KO), and SLC35A2-rescued (A2-KO+A2) HEK293 cells. The deduced structures of the major *N*-glycans are shown. **b**. Extracted ion chromatograms (EICs) of the major complex *N*-glycans shown in Fig. 5B. *N*-Glycans released from desialylated and degalactosylated bovine fetuin were analyzed as standards. EICs of the diagnostic ions for core fucose in MS/MS are also shown. **c**. Comparisons of the LC retention times between samples and standard glycans having defined GlcNAc branches. The standard tri- and tetra-antennary *N*-glycans having different GlcNAc branches were enzymatically formed from the bi- and tri-antennary glycans of bovine fetuin using

purified GnT-III, -IVa, and -V. EICs of the standard glycans with 2, 3, and 4 HexNAc residues (HexNAc in chitobiose is excluded) from LC-MS analysis are shown.

Figure 1

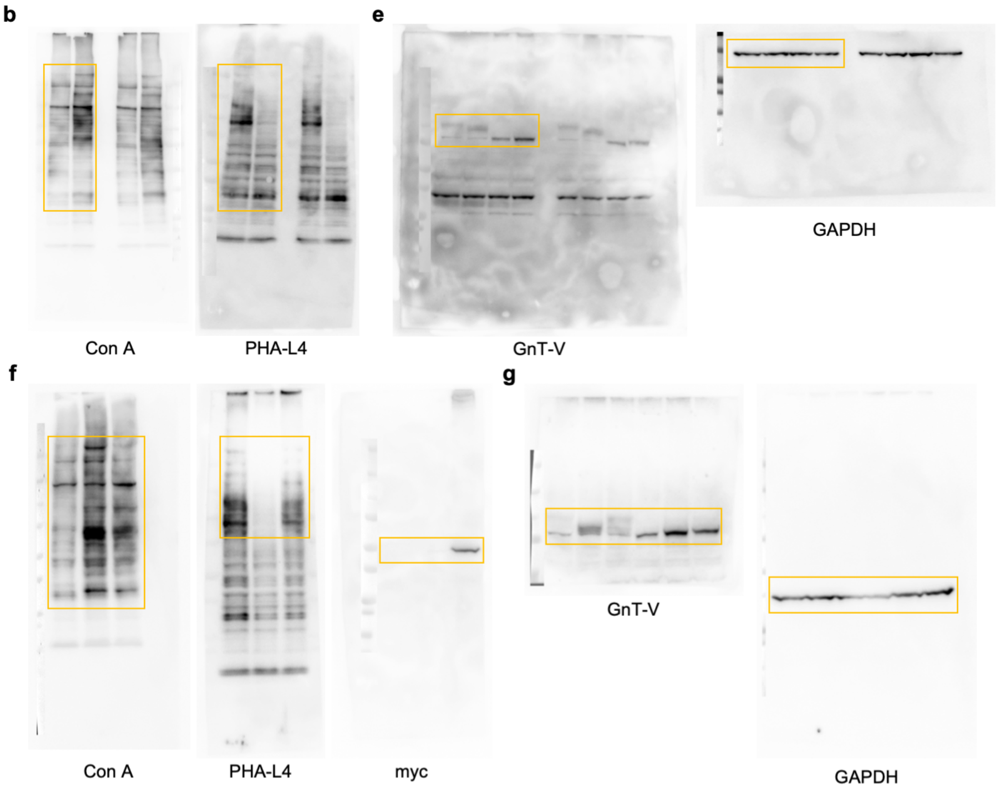

Figure 2

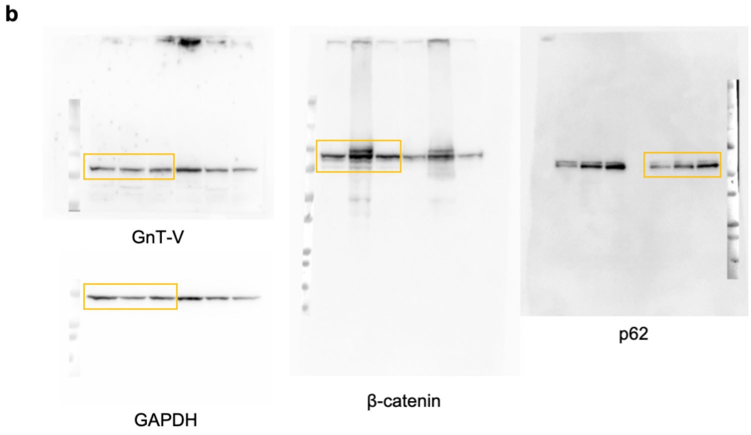

Figure 2

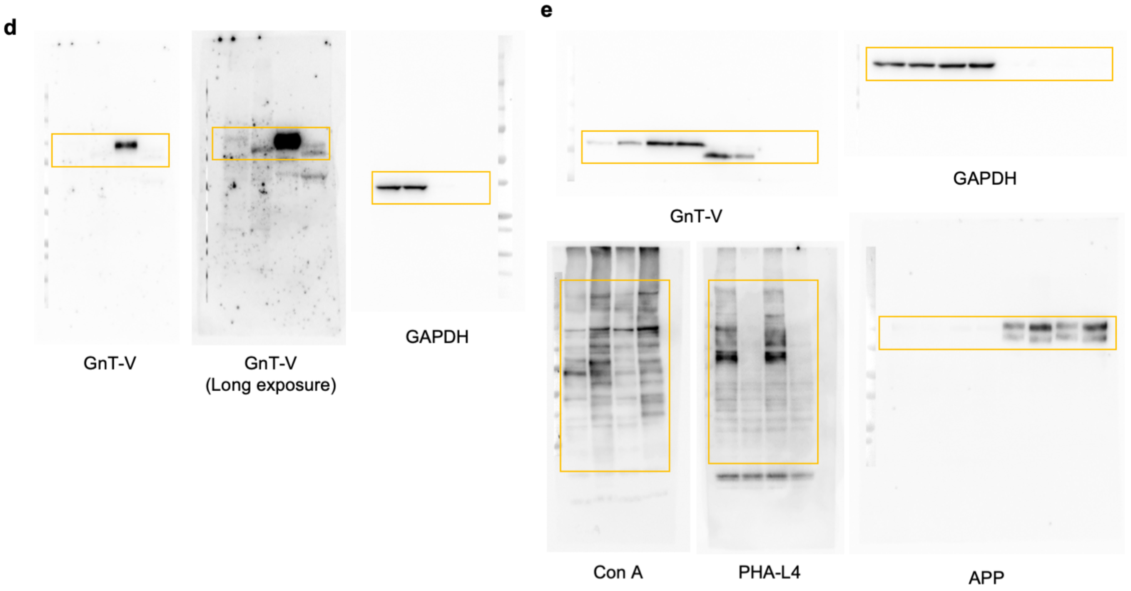

Figure 3

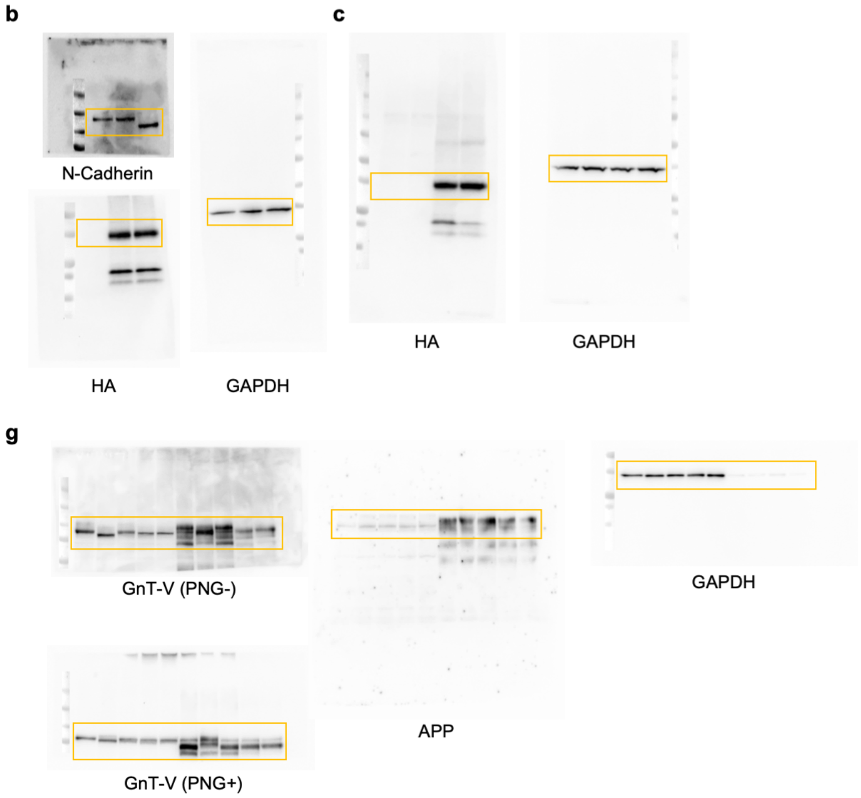

Figure 4

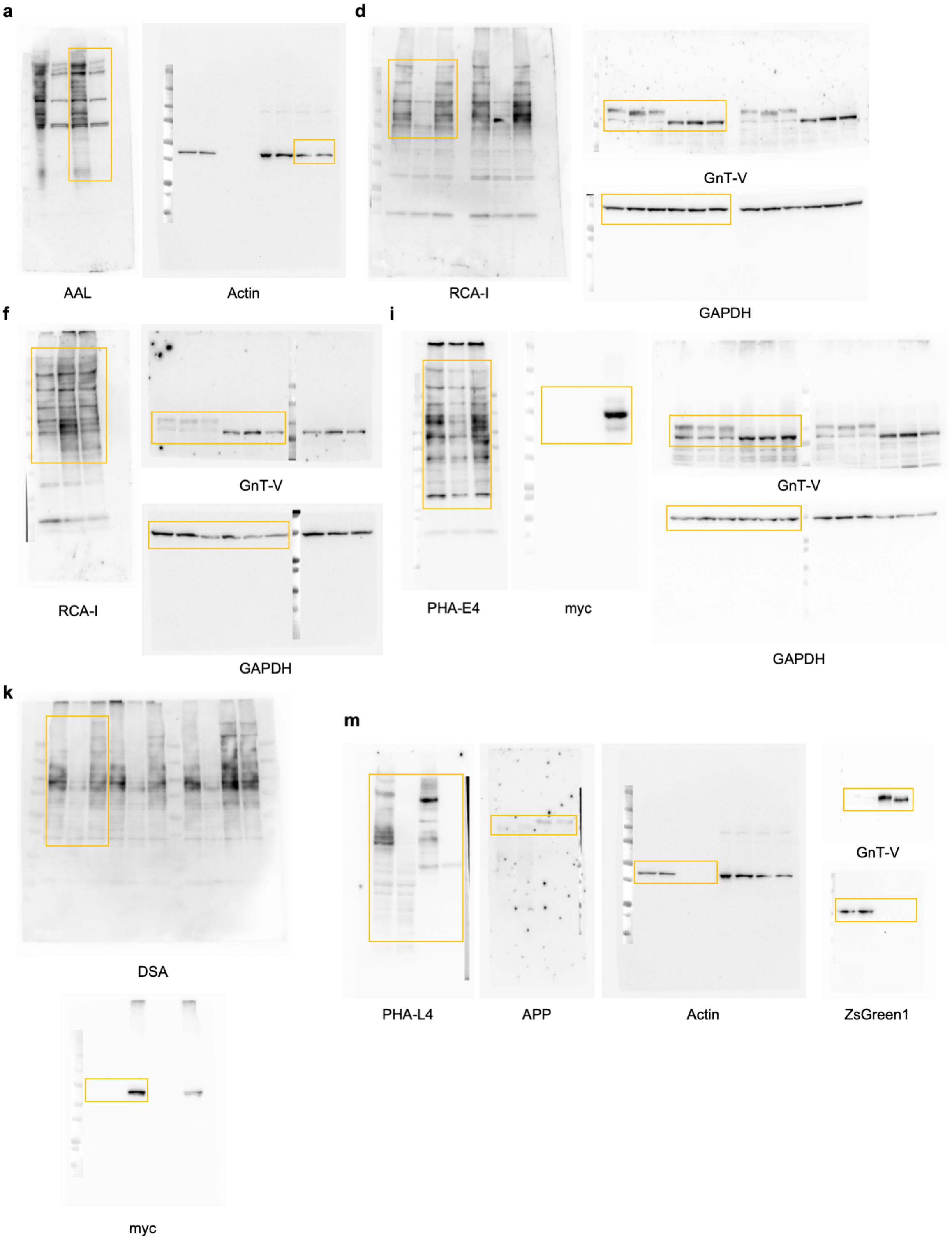
a
AAL
Actin
d
RCA-I
GnT-V
GAPDH
f
RCA-I
GnT-V
GAPDH
i
PHA-E4
myc
GnT-V
GAPDH
k
DSA
myc
m
PHA-L4
APP
Actin
GnT-V
ZsGreen1

Figure 6

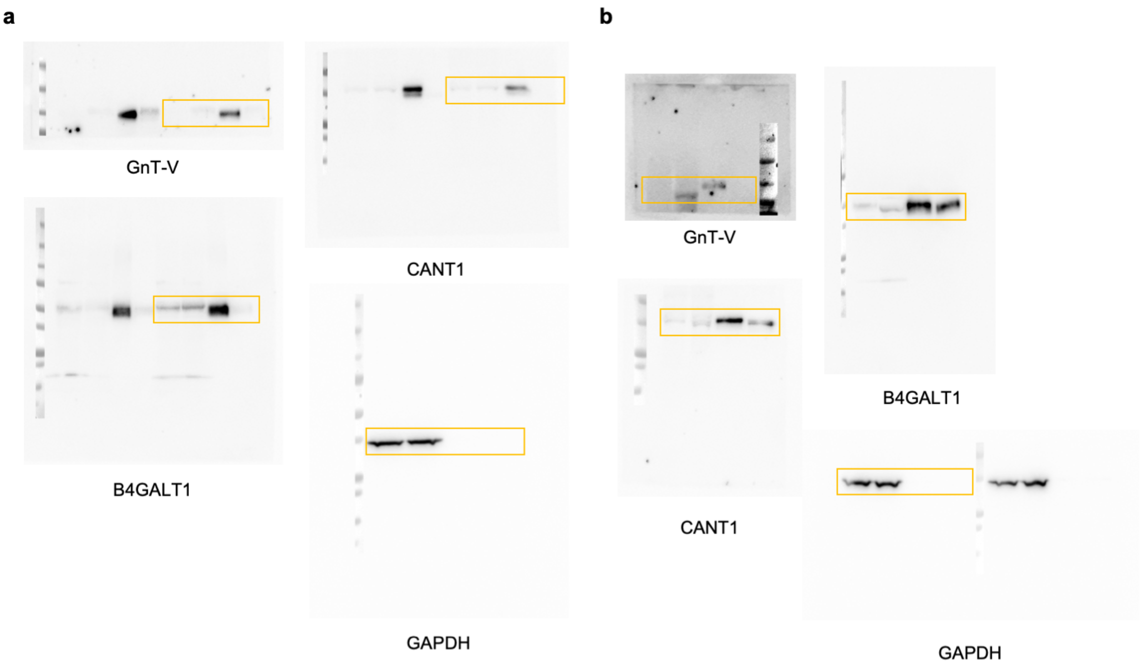

Supplementary Figure 3

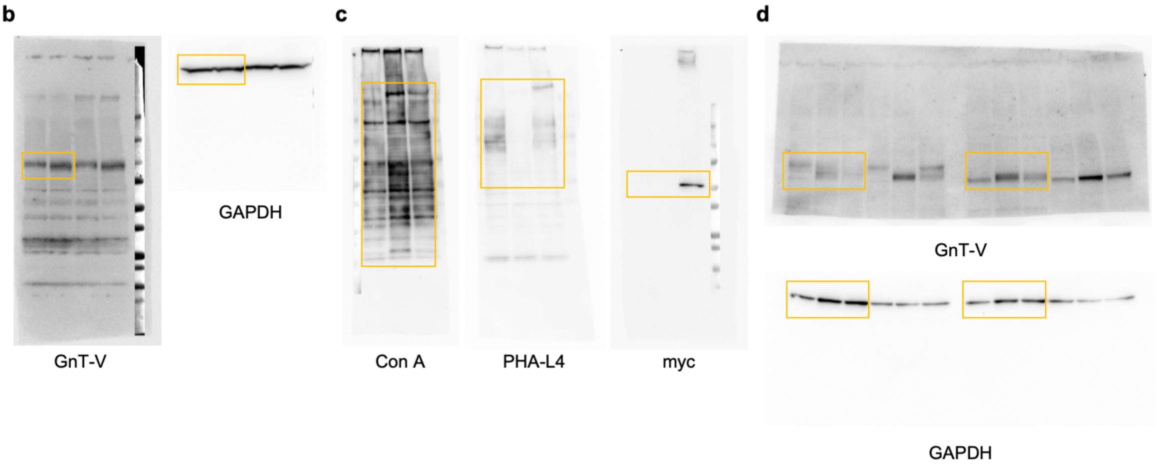

Supplementary Figure 4

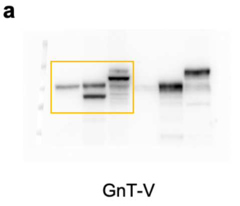

Supplementary Figure 6

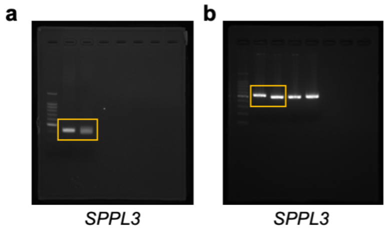

Supplementary Figure 8

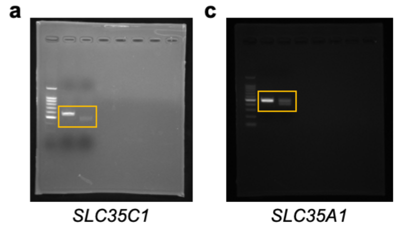

Supplementary Figure 9

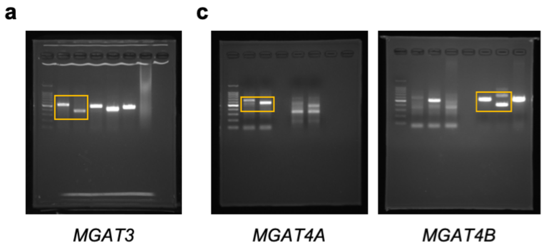

Supplementary Figure 11. Uncropped images of membranes and gels
