# Supplementary material for: Shedding of N-acetylglucosaminyltransferase-V is regulated by maturity of cellular N-glycan
Source: Commun Biol. 2022 Aug 1;5:743. doi: 10.1038/s42003-022-03697-y (PMC9343384; doi:10.1038/s42003-022-03697-y)
Supplement: Supplementary file 3 — Description of Additional Supplementary Files [file 42003_2022_3697_MOESM3_ESM.pdf]

Description of Additional Supplementary Files

File name: Supplementary Data 1
Description: Primers used in this study.

File name: Supplementary Data 2
Description: Intensity of all N-glycans detected in LC-MS.

File name: Supplementary Data 3
Description: The source data behind the graphs in the paper.
