# Supplementary material for: Shedding of N-acetylglucosaminyltransferase-V is regulated by maturity of cellular N-glycan
Source: Commun Biol. 2022 Aug 1;5:743. doi: 10.1038/s42003-022-03697-y (PMC9343384; doi:10.1038/s42003-022-03697-y)
Supplement: Supplementary file 7 — Reporting Summary [file 42003_2022_3697_MOESM7_ESM.pdf]

nature portfolio

Corresponding author(s): Yasuhiko Kizuka

Last updated by author(s): Jun 14, 2022

Reporting Summary

Nature Portfolio wishes to improve the reproducibility of the work that we publish. This form provides structure for consistency and transparency in reporting. For further information on Nature Portfolio policies, see our Editorial Policies and the Editorial Policy Checklist.

Statistics

For all statistical analyses, confirm that the following items are present in the figure legend, table legend, main text, or Methods section.

n/a	Confirmed	
☐	☒	The exact sample size (*n*) for each experimental group/condition, given as a discrete number and unit of measurement
☐	☒	A statement on whether measurements were taken from distinct samples or whether the same sample was measured repeatedly
☐	☒	The statistical test(s) used AND whether they are one- or two-sided<br>*Only common tests should be described solely by name; describe more complex techniques in the Methods section.*
☒	☐	A description of all covariates tested
☐	☒	A description of any assumptions or corrections, such as tests of normality and adjustment for multiple comparisons
☐	☒	A full description of the statistical parameters including central tendency (e.g. means) or other basic estimates (e.g. regression coefficient) AND variation (e.g. standard deviation) or associated estimates of uncertainty (e.g. confidence intervals)
☐	☒	For null hypothesis testing, the test statistic (e.g. *F*, *t*, *r*) with confidence intervals, effect sizes, degrees of freedom and *P* value noted<br>*Give P values as exact values whenever suitable.*
☒	☐	For Bayesian analysis, information on the choice of priors and Markov chain Monte Carlo settings
☒	☐	For hierarchical and complex designs, identification of the appropriate level for tests and full reporting of outcomes
☒	☐	Estimates of effect sizes (e.g. Cohen's *d*, Pearson's *r*), indicating how they were calculated

Our web collection on statistics for biologists contains articles on many of the points above.

Software and code

Policy information about availability of computer code

Data collection	LCSolution, BZ-X800 viewer, EvolutionCapt, Bio-Rad CFX Maestro ver. 1.1, FACSChorus, Xcalibur software ver. 2.2
Data analysis	LCSolution, BZ-X800 analyzer, EvolutionCapt, Bio-Rad CFX Maestro ver. 1.1, FlowJo software (10.8.0), GlycoMod software (https://web.expasy.org/glycomod/), GraphPad Prism 8 software

For manuscripts utilizing custom algorithms or software that are central to the research but not yet described in published literature, software must be made available to editors and reviewers. We strongly encourage code deposition in a community repository (e.g. GitHub). See the Nature Portfolio guidelines for submitting code & software for further information.

Data

Policy information about availability of data

All manuscripts must include a data availability statement. This statement should provide the following information, where applicable:

- Accession codes, unique identifiers, or web links for publicly available datasets
- A description of any restrictions on data availability
- For clinical datasets or third party data, please ensure that the statement adheres to our policy

LC-MS data of N-glycomics in HEK293-SLC35A2-KO cells has been deposited in GlycoPOST under announced ID: GPST000216 (URL: https://glycopost.glycosmos.org/preview/205327909613f2acfd783f; PIN CODE: 7554).

Field-specific reporting

Please select the one below that is the best fit for your research. If you are not sure, read the appropriate sections before making your selection.

☒ Life sciences ☐ Behavioural & social sciences ☐ Ecological, evolutionary & environmental sciences

For a reference copy of the document with all sections, see nature.com/documents/nr-reporting-summary-flat.pdf

Life sciences study design

All studies must disclose on these points even when the disclosure is negative.

Sample size	We initially performed three independent experiments and determined whether additional experiments were required considering the tendency.
Data exclusions	No data were excluded from this study.
Replication	All attempts to replication were successful.
Randomization	This is not relevant to this study because the paper does not include comparison studies among group.
Blinding	The investigators were not blinded during data collection.

Reporting for specific materials, systems and methods

We require information from authors about some types of materials, experimental systems and methods used in many studies. Here, indicate whether each material, system or method listed is relevant to your study. If you are not sure if a list item applies to your research, read the appropriate section before selecting a response.

Materials & experimental systems

n/a	Involved in the study	
☐	☒	Antibodies
☐	☒	Eukaryotic cell lines
☒	☐	Palaeontology and archaeology
☒	☐	Animals and other organisms
☒	☐	Human research participants
☒	☐	Clinical data
☒	☐	Dual use research of concern

Methods

n/a	Involved in the study	
☒	☐	ChIP-seq
☐	☒	Flow cytometry
☒	☐	MRI-based neuroimaging

Antibodies

Antibodies used	The following antibodies were used in this study: anti-GnT-V monoclonal antibody [mAb; clone 24D11 (Murata et al., 2000); a gift from Dr. Eiji Miyoshi (Osaka University, Osaka, Japan)]; anti-GAPDH mAb (clone 6C5), anti-Amyloid precursor protein (APP) (clone 22C11), anti-actin mAb (clone AC-40), and anti-myc-tag mAb (clone 4A6) from Millipore (MAB374, MAB348, A4700, and 05-724); anti-beta-catenin mAb (clone 14) and anti-GM130 mAb (clone 35) from BD Biosciences (610154 and 610822); rabbit anti-Sequestosome 1 (SQSTM1)/p62 and rabbit anti-HA-tag mAb (clone C29F4) from Cell Signaling Technology (5114 and 3724); anti-GnT-V mAb (clone 706824), anti-CANT1 mAb (clone 861206), and goat anti-B4GALT1 from R and D systems (MAB5469, MAB6720, and AF3609); anti-FLAG mAb (clone M2) from Sigma-Aldrich (F1804); anti-ZsGreen1 mAb (clone 2C2) from ORIGENE; rabbit anti-calnexin (ab22595) from Abcam; rat anti-HA (clone 3F10) from Merck (11867423001); horseradish peroxidase (HRP)-conjugated anti-mouse immunoglobulin (Ig) G and HRP-conjugated anti-rabbit IgG from GE Healthcare (NA931V and NA934V); HRP-conjugated anti-goat IgG from Jackson ImmunoResearch Laboratories (705-035-147); and Alexa488-conjugated anti-mouse IgG, Alexa488-conjugated anti-rabbit IgG, Alexa546-conjugated anti-mouse IgG, and Alexa546-conjugated anti-rat IgG from ThermoFisher Scientific (A21202, A21206, A10036, and A11081).
Validation	anti-GnT-V from Dr. Miyoshi and R and D systems were validated by GnT-V KO cells. The validation of all other antibodies were available from each web site.

Eukaryotic cell lines

Policy information about cell lines

Cell line source(s)	HEK293, Neuro-2A, GnT-I-deficient HEK293S (Reeves et al., 2002), HeLa, CHO, and Lec1 (Stanley et al., 1975b) cells were obtained from the American Type Culture Collection, and COS7 cells and B16 cells were purchased from RIKEN cell bank.

Authentication	None of the cell lines were authenticated.
Mycoplasma contamination	All cell lines were not tested for mycoplasma contaminination.
Commonly misidentified lines (See ICLAC register)	HeLa cells and CHO cells were listed while other cell lines were not.

Flow Cytometry

Plots

Confirm that:

☒ The axis labels state the marker and fluorochrome used (e.g. CD4-FITC).

☒ The axis scales are clearly visible. Include numbers along axes only for bottom left plot of group (a 'group' is an analysis of identical markers).

☒ All plots are contour plots with outliers or pseudocolor plots.

☒ A numerical value for number of cells or percentage (with statistics) is provided.

Methodology

Sample preparation	Cells were washed with PBS twice and collected using cell scrapers, followed by centrifugation at 1,400 x g for 3 min. The cells were washed with FACS buffer (1% BSA, 0.1% NaN3, in PBS) once and stained with fluorescently-labeled lectin in FACS buffer on ice for 15 min. For use of biotinylated lectins, the cells were first incubated with biotinylated lectin in FACS buffer on ice for 15 min, followed by incubation with fluorescently-labeled streptavidin in FACS buffer on ice for 15 min.
Instrument	FACS Melody cell sorter (BD)
Software	Data were collected by FACSChorus software and analyzed using FlowJo software (10.8.0).
Cell population abundance	We collected 30,000 cells for each samples in which more than 50% was analyzed as viable cells.
Gating strategy	We used cell lines and gating of different populations does not apply to this study.

☐ Tick this box to confirm that a figure exemplifying the gating strategy is provided in the Supplementary Information.
